# Supplementary material for: Transcriptome-informed brain cartography of polygenic risk and association with brain structure in major psychiatric disorders
Source: Mol Psychiatry. 2026 Mar 6;31(7):3965–77. doi: 10.1038/s41380-026-03497-4 (PMC13268975; doi:10.1038/s41380-026-03497-4)
Supplement: Supplementary file 1 — Supplementary_Transcriptomic_informed_brain_cartography [file 41380_2026_3497_MOESM1_ESM.docx]

**Supplementary Materials: Transcriptome-informed brain cartography of polygenic risk and association with brain structure in major psychiatric disorders**

Alessio Giacomel^1,2,3^, Timothy R. Powell^4^, Rodrigo R. R. Duarte^4,5^, Giovanna Nordio^1^, Steve C. R. Williams^1^, Federico Turkheimer^1,6^, Mattia Veronese^1,7^, Daniel Martins^1,8,9*^, Danai Dima^1,10,*^

^1^ *Department of Neuroimaging, Institute of Psychiatry, Psychology and Neuroscience, King's College London, London, UK*

*^2^ Department of Child and Adolescent Psychiatry, University Hospital, Goethe University, Frankfurt am Main, Germany*

*^3^ Cooperative Brain Imaging Centre (COBIC), Goethe University, Frankfurt am Main, Germany*

*^4^ Social, Genetic & Developmental Psychiatry Centre, Institute of Psychiatry, Psychology & Neuroscience, King’s College London, London, UK*

*^5^ Department of Medical & Molecular Genetics, Faculty of Life Sciences & Medicine, King’s College London, London, UK*

*^6^The Institute for Human and Synthetic Minds, King's College London, London, UK*

*^7^ Department of Information Engineering, University of Padova, Padova, Italy*

*^8^ Department of Clinical Neurosciences and Mental Health, Faculty of Medicine, University of Porto, Portugal*

*^9^ RISE-Health Network (Neurosciences thematic line), Faculty of Medicine, University of Porto, Portugal*

*^10^ Department of Psychology and Neuroscience, School of Health and Medical Sciences, City St George’s, University of London, London, UK*

^*^ *Equal contribution*

**Corresponding author:**

Alessio Giacomel

Email: Alessio.giacomel@kcl.ac.uk

Department of Neuroimaging

Centre for Neuroimaging Sciences

Institute of Psychiatry, Psychology and Neuroscience, King’s College London

Denmark Hill, SE5 8AF, London, UK

Phone:

**Preliminary analyses with H-MAGMA**

To develop a spatial model of regional variation of structural brain differences from transcriptomic architecture the first step is to convert polygenic risk from the genetic variant space to the gene level space. To achieve this, we used an algorithm of risk calculation at the level of gene such as Multi-marker Analysis of GenoMic Association (MAGMA) ([de Leeuw et al., 2015](https://journals.plos.org/ploscompbiol/article?id=10.1371/journal.pcbi.1004219)).

MAGMA is a widely used tool for gene-based and gene-set association analyses in genome-wide association studies (GWAS). It aggregates SNP-level association statistics into gene-level p-values by applying a multiple linear regression framework that accounts for linkage disequilibrium (LD) and gene size. In our case we performed the analysis using a refined version of MAGMA that is well suited for brain tissues, namely H-MAGMA ([Sey et al., 2020](https://pmc.ncbi.nlm.nih.gov/articles/PMC7131892/)). Unlike traditional MAGMA, which assigns SNPs to genes based on linear proximity, H-MAGMA uses Hi-C chromatin interaction maps to map non-coding variants to their regulatory target genes. This resulted in a list of prioritised genes based on predicted cumulative risk of dysregulation.

We then developed a dysregulation score for each region of the Desikan-Killiany (DK) atlas by combining the regional gene expression pattern, as expressed by the Allen Human Brain Atlas (AHBA), with the weight indicated by H-MAGMA in those genes that showed p-value<0.001. Finally, we correlated these scores with meta-analytical maps of structural brain abnormalities measured by cortical thickness of six psychiatric disorders (ADHD, AN, ASD, BD, MDD, and SCZ) from the Enhancing Imaging Genetics through Meta Analysis (ENIGMA) initiative.

With this approach only one MDD showed significant correlation between H-MAGMA identified genes in the brain and ENIGMA measured regional abnormalities (⍴=0.346, p_spin_=0.042). This approach nonetheless lacked the information about the directionality of the changes. Directionality might play a significant role in psychiatric disorders as some changes might be a protective factor while other might be pathological.

**Supplementary Figure 1. Sampling sites for GTEx prediction models**. The Genotype-Tissue Expression (GTEx) project is a large-scale initiative that characterizes the relationship between genetic variation and gene expression across multiple human tissues. By integrating genomic and transcriptomic data from post-mortem donors, GTEx provides a comprehensive resource for studying tissue-specific gene regulation and identifying expression quantitative trait loci (eQTLs). For brain-related research, GTEx includes RNA-sequencing data from multiple brain regions, such as the cortex, cerebellum, hippocampus, basal ganglia (including the caudate, putamen, and nucleus Accumbens), hypothalamus, amygdala, and substantia nigra. These regions are critical for understanding neurodevelopment, cognition, and neuropsychiatric disorders. GTEx-based elastic net models use cis-genetic variation (within 1 Mb of a gene) to predict gene expression in specific tissues. Elastic net regression combines lasso (L1) and ridge (L2) penalties to select relevant genetic variants while maintaining model stability. In the present study we used the models here described to predict the transcriptomic profile from GWAS data in each tissue and then combine them for a whole brain approach. More information about the GTEx, GTEx panels and methods are available at <https://www.gtexportal.org/home/>.


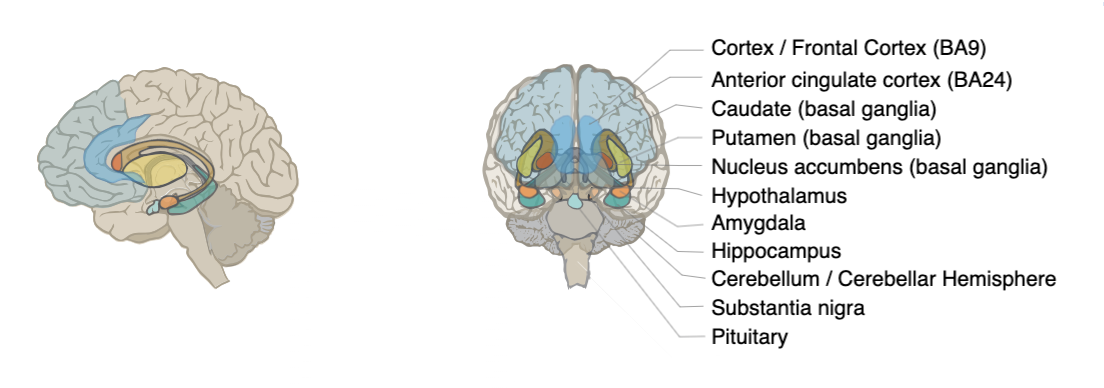


**
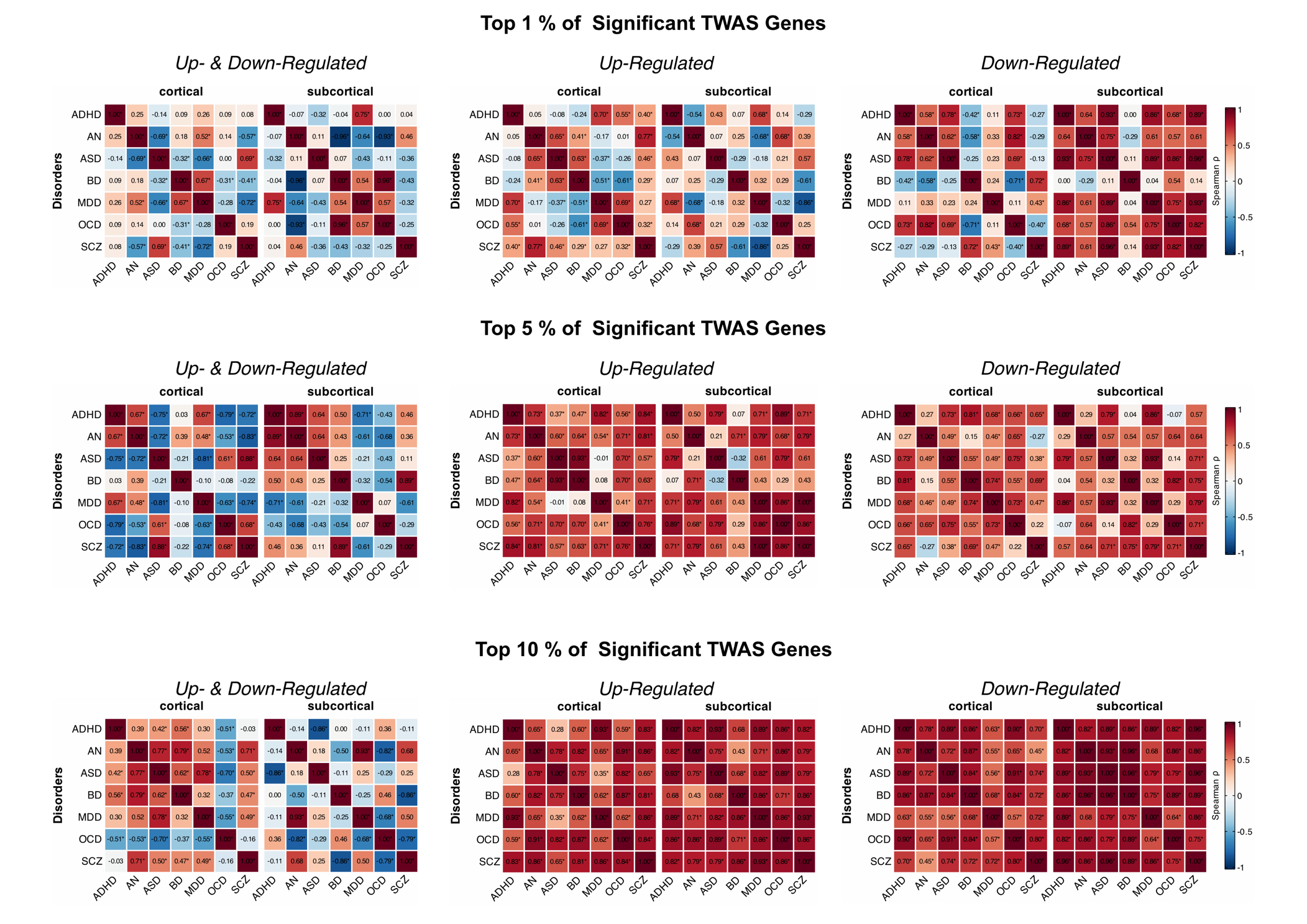
**

**Supplementary Figure 2. Cross Disorder GEDAR Similarity.** Figure shows pairwise Spearman correlation matrices of GEDAR maps across major psychiatric disorders, plotted at three TWAS significance thresholds (top 1%, 5%, and 10% of genes) under up-regulated, down-regulated, and combined weighting schemes. Matrices display similarities for both cortical and subcortical regions of the Desikan-Killiany atlas. The * highlights significant correlations at p_spin_<0.05. Abbreviations: Attention Deficit Hyperactivity Disorder (ADHD), Autism Spectrum Disorder (ASD), Anorexia Nervosa (AN), Bipolar Disorder (BD), Major Depressive Disorder (MDD), Obsessive Compulsive Disorder (OCD), Schizophrenia (SCZ).

**Supplementary Figure 3. Jaccard Similarity of Significant genes.** Figure shows pairwise Jaccard similarity index across psychiatric disorders of top 10, 5, and 1% of genes ranked according to fdr-corrected TWAS p-value. Abbreviations: Attention Deficit Hyperactivity Disorder (ADHD), Autism Spectrum Disorder (ASD), Anorexia Nervosa (AN), Bipolar Disorder (BD), Major Depressive Disorder (MDD), Obsessive Compulsive Disorder (OCD), Schizophrenia (SCZ).


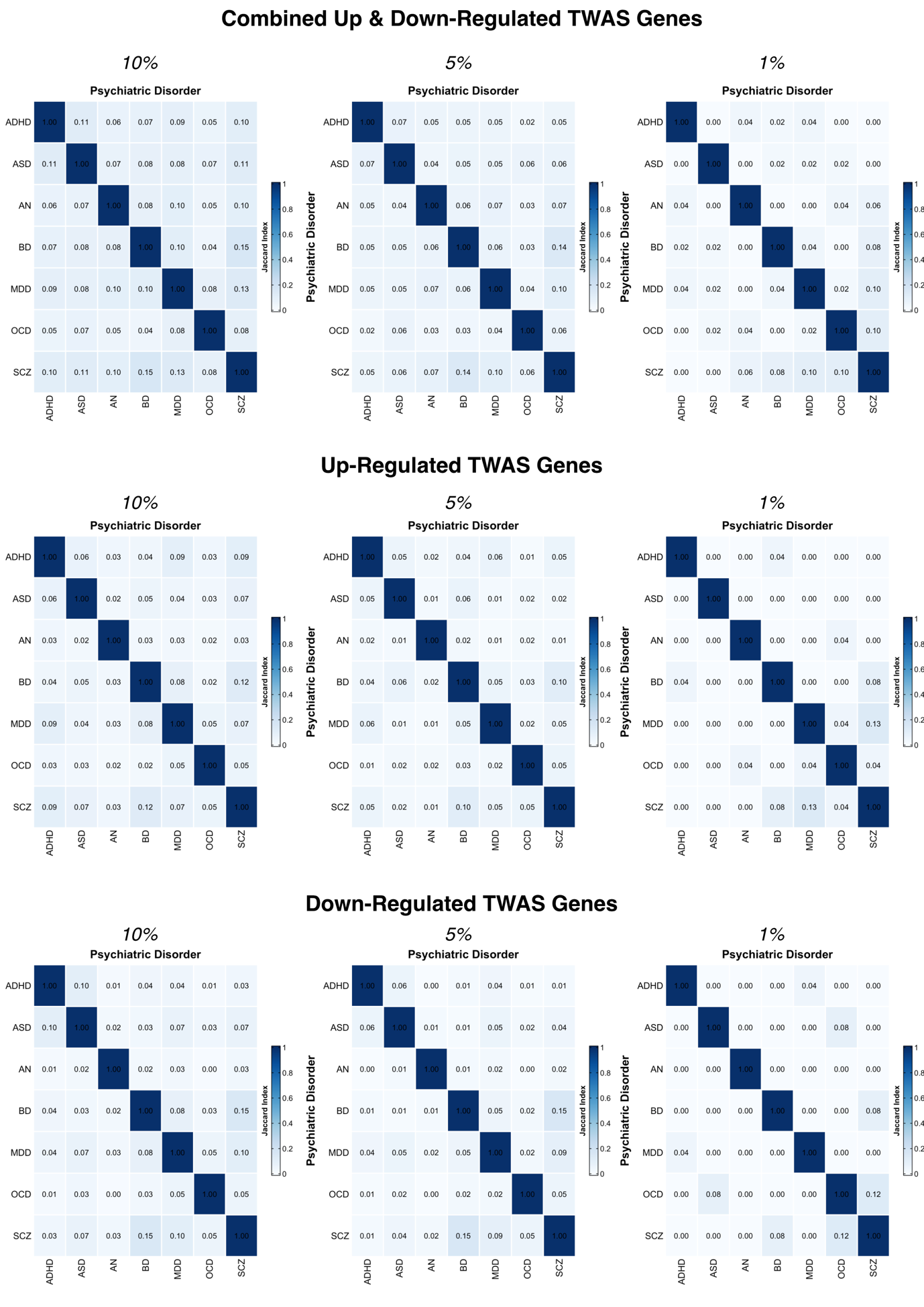


**Supplementary Figure 4. Significant genes across disorders and thresholds.** Figure shows Venn diagrams for the overlap across psychiatric disorders of top 10, 5, and 1% of genes ranked according to FDR-corrected TWAS p-value. Distance between non-overlapping areas is purely aesthetic and is not informative. Abbreviations: Attention Deficit Hyperactivity Disorder (ADHD), Autism Spectrum Disorder (ASD), Anorexia Nervosa (AN), Bipolar Disorder (BD), Major Depressive Disorder (MDD), Obsessive Compulsive Disorder (OCD), Schizophrenia (SCZ).


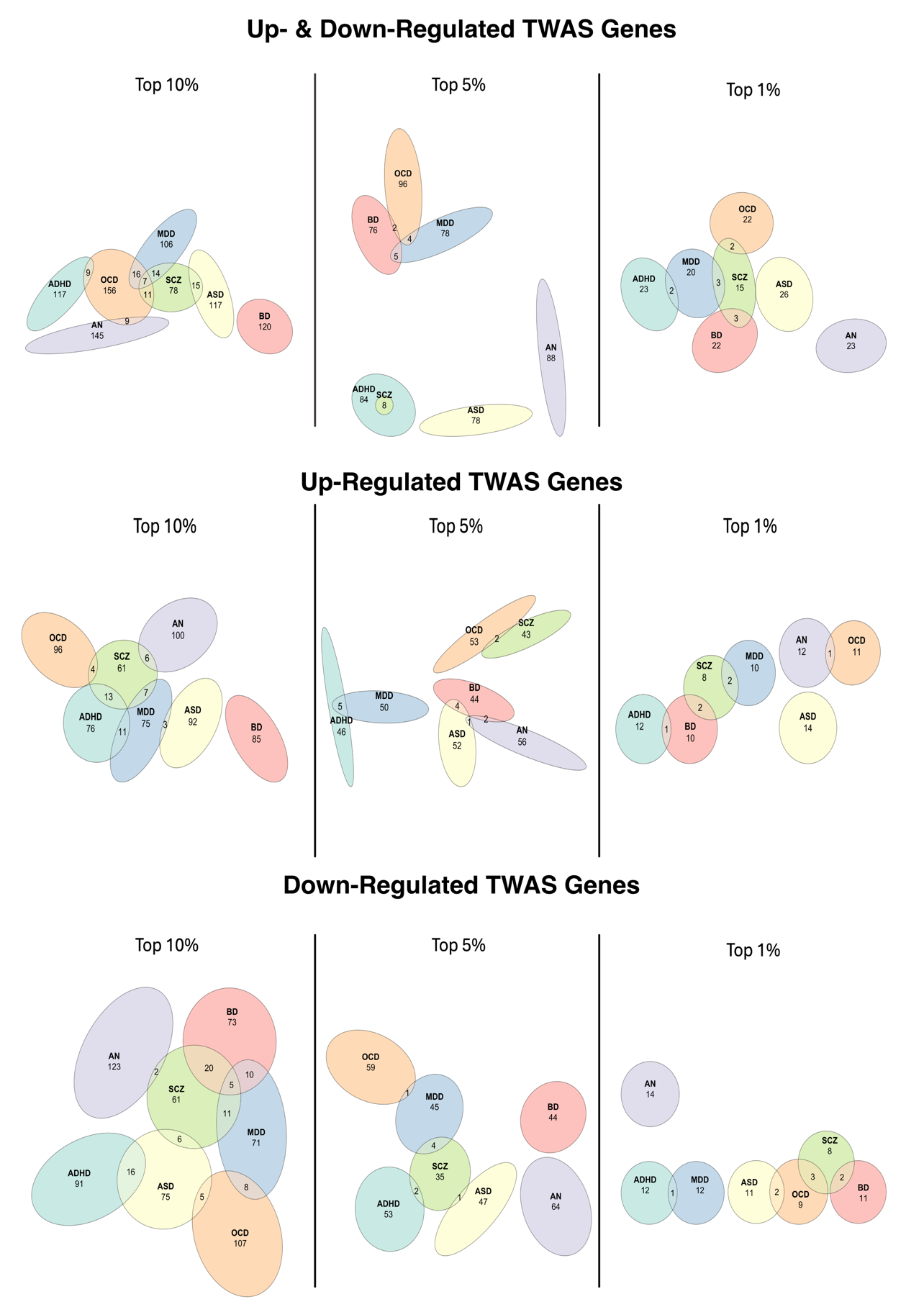


**Supplementary Table S1.** The table shows results for correlation analysis between GEDAR brain maps and brain structural changes. Table shows GEDAR maps for combined up- and down-regulated genes. The * indicates significant p<0.05.

| **Disorder** | **Threshold** | **Compartment** | **Spearman correlation** | **P_spin_-value** | **FDR-corrected p_spin_-value** |
| --- | --- | --- | --- | --- | --- |
| ADHD | 1 | Cortical | 0.130 | 0.274 | 0.274 |
|  |  | Subcortical | -0.396 | 0.181 | 0.271 |
|  | 5 | Cortical | 0.151 | 0.273 | 0.274 |
|  |  | Subcortical | 0.054 | 0.470 | 0.470 |
|  | 10 | Cortical | 0.252 | 0.131 | 0.274 |
|  |  | Subcortical | 0.920 | 0.002** | 0.006** |
| AN | 1 | Cortical | -0.517 | 0.056 | 0.122 |
|  |  | Subcortical | 0.214 | 0.349 | 0.418 |
|  | 5 | Cortical | -0.437 | 0.098 | 0.122 |
|  |  | Subcortical | 0.321 | 0.240 | 0.418 |
|  | 10 | Cortical | -0.408 | 0.122 | 0.122 |
|  |  | Subcortical | 0.071 | 0.418 | 0.418 |
| ASD | 1 | Cortical | -0.150 | 0.252 | 0.378 |
|  |  | Subcortical | -0.262 | 0.289 | 0.316 |
|  | 5 | Cortical | 0.413 | 0.073 | 0.219 |
|  |  | Subcortical | 0.206 | 0.316 | 0.316 |
|  | 10 | Cortical | -0.053 | 0.423 | 0.423 |
|  |  | Subcortical | 0.449 | 0.154 | 0.316 |
| BD | 1 | Cortical | 0.175 | 0.153 | 0.153 |
|  |  | Subcortical | 0.214 | 0.303 | 0.303 |
|  | 5 | Cortical | 0.456 | 0.01** | 0.03* |
|  |  | Subcortical | -0.321 | 0.229 | 0.303 |
|  | 10 | Cortical | 0.314 | 0.09 | 0.135 |
|  |  | Subcortical | -0.571 | 0.089 | 0.392 |
| MDD | 1 | Cortical | 0.531 | 0.003** | 0.0045** |
|  |  | Subcortical | 0.571 | 0.083 | 0.136 |
|  | 5 | Cortical | 0.547 | 0.003** | 0.0045** |
|  |  | Subcortical | 0.571 | 0.091 | 0.136 |
|  | 10 | Cortical | 0.529 | 0.005** | 0.005** |
|  |  | Subcortical | -0.143 | 0.392 | 0.392 |
| OCD | 1 | Cortical | -0.191 | 0.111 | 0.166 |
|  |  | Subcortical | 0.214 | 0.311 | 0.311 |
|  | 5 | Cortical | -0.097 | 0.266 | 0.266 |
|  |  | Subcortical | -0.250 | 0.283 | 0.311 |
|  | 10 | Cortical | -0.211 | 0.086 | 0.166 |
|  |  | Subcortical | -0.429 | 0.161 | 0.311 |
| SCZ | 1 | Cortical | -0.101 | 0.331 | 0.486 |
|  |  | Subcortical | -0.288 | 0.242 | 0.242 |
|  | 5 | Cortical | -0.002 | 0.486 | 0.486 |
|  |  | Subcortical | -0.667 | 0.047* | 0.070 |
|  | 10 | Cortical | -0.333 | 0.057 | 0.171 |
|  |  | Subcortical | 0.702 | 0.041* | 0.070 |

**Supplementary Figure 5. ENIGMA-GEDAR correlation.** Figure depicts spearman correlations between ENIGMA maps of structural differences and GEDAR maps calculated at the three different thresholds separately for cortical and subcortical regions. Significance was calculated by generating 1000 random spin permutations to account for spatial autocorrelation after FDR correction for multiple comparisons across disorders. The * highlights significant correlations at p_spin-FDR_<0.05. Abbreviations: Attention Deficit Hyperactivity Disorder (ADHD), Autism Spectrum Disorder (ASD), Anorexia Nervosa (AN), Bipolar Disorder (BD), Major Depressive Disorder (MDD), Obsessive Compulsive Disorder (OCD), Schizophrenia (SCZ).


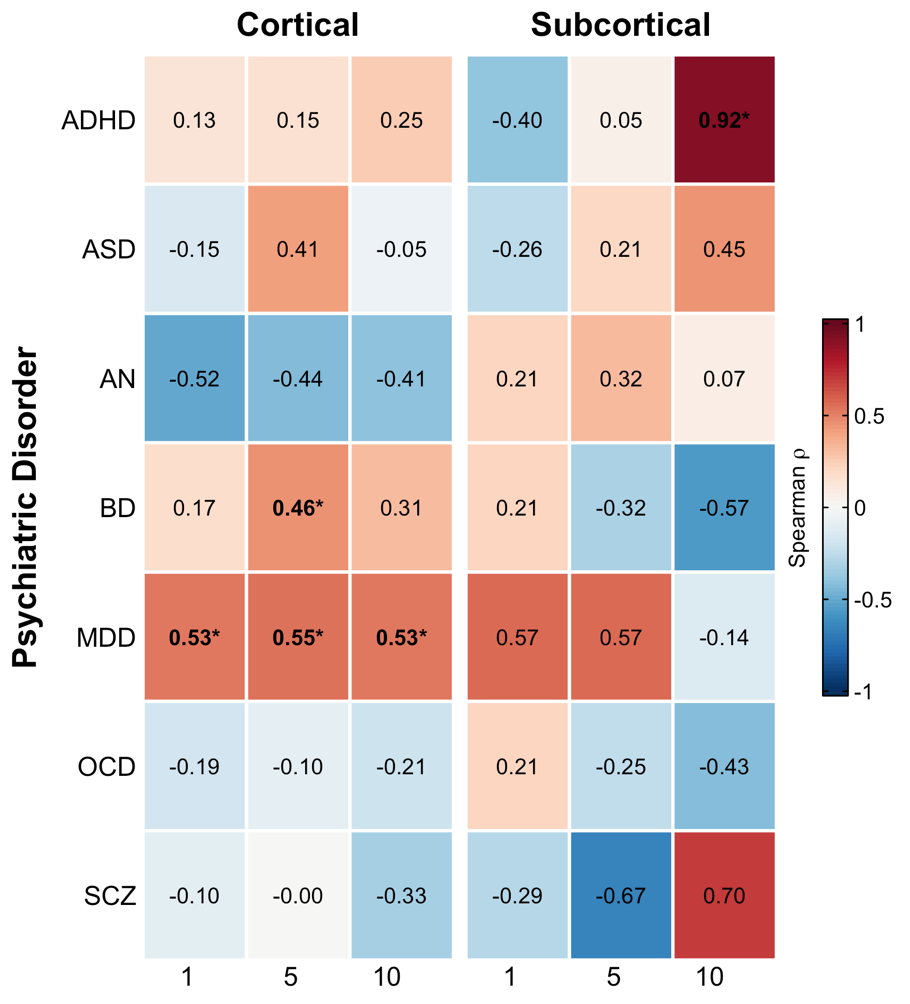


**Supplementary Table S2.** The table shows results for correlation analysis between GEDAR brain maps and brain structural changes. Table shows GEDAR maps for up-regulated genes. The * indicates significant p<0.05.

| **Disorder** | **Threshold** | **Compartment** | **Spearman correlation** | **p-value** | **FDR-corrected p_spin_-value** |
| --- | --- | --- | --- | --- | --- |
| ADHD | 1 | Cortical | 0.024 | 0.458 | 0.458 |
|  |  | Subcortical | -0.775 | 0.029* | 0.087 |
|  | 5 | Cortical | -0.178 | 0.235 | 0.409 |
|  |  | Subcortical | 0.036 | 0.484 | 0.484 |
|  | 10 | Cortical | -0.116 | 0.273 | 0.409 |
|  |  | Subcortical | -0.144 | 0.347 | 0.484 |
| AN | 1 | Cortical | 0.223 | 0.125 | 0.249 |
|  |  | Subcortical | -0.143 | 0.375 | 0.468 |
|  | 5 | Cortical | -0.202 | 0.166 | 0.249 |
|  |  | Subcortical | 0.000 | 0.468 | 0.468 |
|  | 10 | Cortical | -0.012 | 0.512 | 0.512 |
|  |  | Subcortical | 0.107 | 0.422 | 0.468 |
| ASD | 1 | Cortical | -0.095 | 0.35 | 0.340 |
|  |  | Subcortical | -0.486 | 0.131 | 0.393 |
|  | 5 | Cortical | 0.092 | 0.340 | 0.340 |
|  |  | Subcortical | 0.037 | 0.476 | 0.476 |
|  | 10 | Cortical | -0.093 | 0.416 | 0.340 |
|  |  | Subcortical | 0.449 | 0.154 | 0.476 |
| BD | 1 | Cortical | 0.001 | 0.524 | 0.524 |
|  |  | Subcortical | 0.393 | 0.184 | 0.276 |
|  | 5 | Cortical | -0.195 | 0.142 | 0.370 |
|  |  | Subcortical | -0.143 | 0.376 | 0.376 |
|  | 10 | Cortical | -0.126 | 0.247 | 0.370 |
|  |  | Subcortical | -0.428 | 0.163 | 0.276 |
| MDD | 1 | Cortical | 0.396 | 0.031* | 0.093 |
|  |  | Subcortical | 0.143 | 0.376 | 0.376 |
|  | 5 | Cortical | 0.257 | 0.131 | 0.196 |
|  |  | Subcortical | -0.464 | 0.135 | 0.271 |
|  | 10 | Cortical | -0.028 | 0.404 | 0.404 |
|  |  | Subcortical | -0.428 | 0.181 | 0.271 |
| OCD | 1 | Cortical | 0.168 | 0.125 | 0.349 |
|  |  | Subcortical | -0.178 | 0.321 | 0.329 |
|  | 5 | Cortical | -0.025 | 0.440 | 0.440 |
|  |  | Subcortical | -0.607 | 0.060 | 0.180 |
|  | 10 | Cortical | -0.128 | 0.233 | 0.349 |
|  |  | Subcortical | -0.500 | 0.124 | 0.186 |
| SCZ | 1 | Cortical | -0.009 | 0.490 | 0.490 |
|  |  | Subcortical | -0.342 | 0.212 | 0.212 |
|  | 5 | Cortical | 0.025 | 0.438 | 0.490 |
|  |  | Subcortical | -0.541 | 0.101 | 0.212 |
|  | 10 | Cortical | -0.05 | 0.360 | 0.490 |
|  |  | Subcortical | -0.396 | 0.194 | 0.212 |

**Supplementary Figure 6. ENIGMA-GEDAR correlation.** Figure depicts spearman correlations between ENIGMA maps of structural differences and GEDAR maps calculated at the three different thresholds separately for cortical and subcortical regions. Significance was calculated by generating 1000 random spin permutations to account for spatial autocorrelation after FDR correction for multiple comparisons across disorders. The * highlights significant correlations at p_spin-FDR_<0.05. Abbreviations: Attention Deficit Hyperactivity Disorder (ADHD), Autism Spectrum Disorder (ASD), Anorexia Nervosa (AN), Bipolar Disorder (BD), Major Depressive Disorder (MDD), Obsessive Compulsive Disorder (OCD), Schizophrenia (SCZ).

**
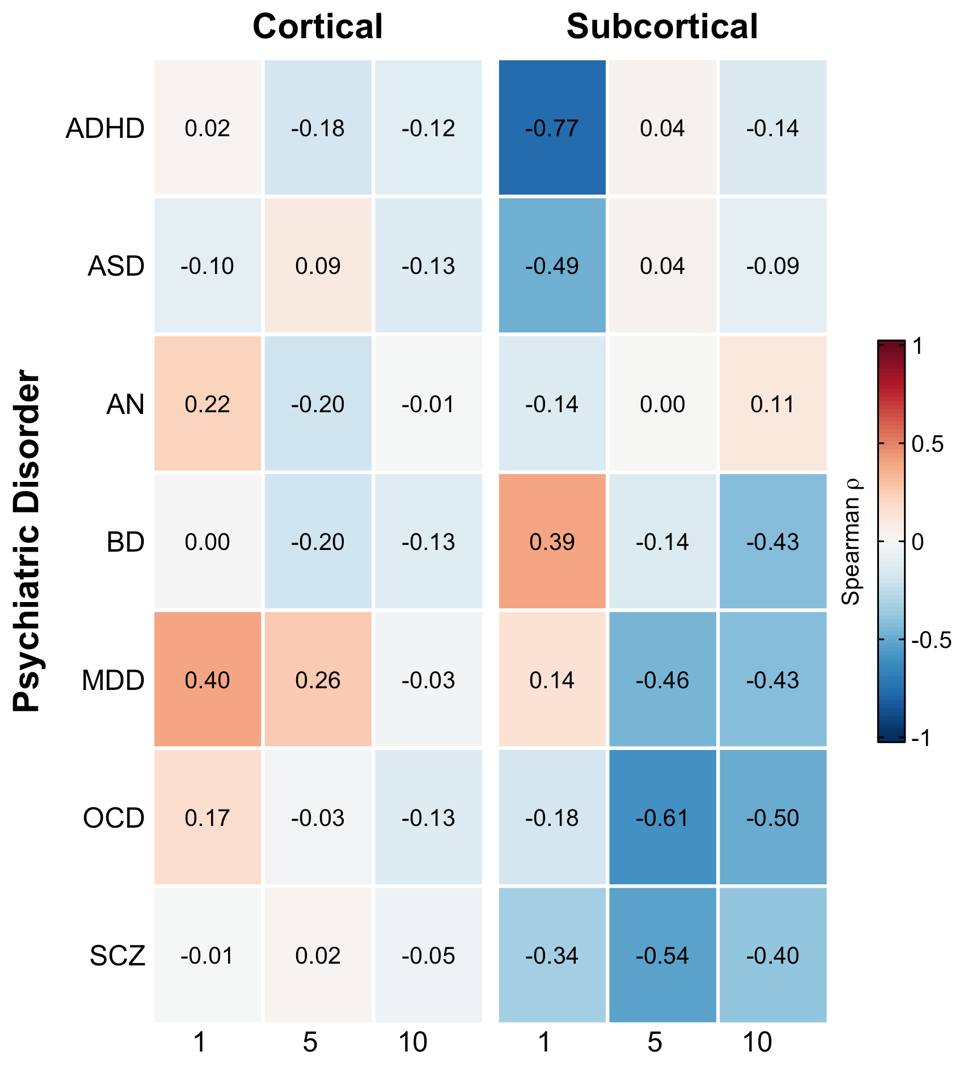
**

**Supplementary Table S3.** The table shows results for correlation analysis between GEDAR brain maps and brain structural changes. Table shows GEDAR maps for down-regulated genes. The * indicates significant p<0.05.

| **Disorder** | **Threshold** | **Compartment** | **Spearman correlation** | **p-value** | **FDR-corrected p_spin_-value** |
| --- | --- | --- | --- | --- | --- |
| ADHD | 1 | Cortical | -0.045 | 0.412 | 0.412 |
|  |  | Subcortical | -0.198 | 0.319 | 0.476 |
|  | 5 | Cortical | -0.401 | 0.009** | 0.027* |
|  |  | Subcortical | 0.000 | 0.476 | 0.476 |
|  | 10 | Cortical | -0.300 | 0.049* | 0.073 |
|  |  | Subcortical | -0.144 | 0.347 | 0.476 |
| AN | 1 | Cortical | -0.348 | 0.124 | 0.186 |
|  |  | Subcortical | 0.214 | 0.349 | 0.228 |
|  | 5 | Cortical | -0.452 | 0.083 | 0.186 |
|  |  | Subcortical | 0.321 | 0.240 | 0.228 |
|  | 10 | Cortical | -0.252 | 0.211 | 0.211 |
|  |  | Subcortical | 0.071 | 0.418 | 0.285 |
| ASD | 1 | Cortical | 0.238 | 0.316 | 0.498 |
|  |  | Subcortical | 0.280 | 0.271 | 0.442 |
|  | 5 | Cortical | 0.082 | 0.411 | 0.498 |
|  |  | Subcortical | -0.056 | 0.442 | 0.442 |
|  | 10 | Cortical | 0.001 | 0.498 | 0.498 |
|  |  | Subcortical | -0.093 | 0.425 | 0.442 |
| BD | 1 | Cortical | 0.033 | 0.439 | 0.493 |
|  |  | Subcortical | -0.250 | 0.287 | 0.287 |
|  | 5 | Cortical | 0.168 | 0.185 | 0.277 |
|  |  | Subcortical | -0.464 | 0.133 | 0.243 |
|  | 10 | Cortical | 0.209 | 0.132 | 0.277 |
|  |  | Subcortical | -0.428 | 0.162 | 0.243 |
| MDD | 1 | Cortical | -0.278 | 0.066 | 0.099 |
|  |  | Subcortical | -0.357 | 0.214 | 0.249 |
|  | 5 | Cortical | -0.201 | 0.144 | 0144 |
|  |  | Subcortical | -0.500 | 0.131 | 0.249 |
|  | 10 | Cortical | -0.424 | 0.017* | 0.051 |
|  |  | Subcortical | -0.286 | 0.249 | 0.249 |
| OCD | 1 | Cortical | 0.207 | 0.088 | 0.187 |
|  |  | Subcortical | -0.321 | 0.240 | 0.326 |
|  | 5 | Cortical | 0.189 | 0.125 | 0.187 |
|  |  | Subcortical | -0.250 | 0.288 | 0.326 |
|  | 10 | Cortical | 0.046 | 0.408 | 0.408 |
|  |  | Subcortical | -0.214 | 0.326 | 0.326 |
| SCZ | 1 | Cortical | -0.032 | 0.445 | 0.445 |
|  |  | Subcortical | -0.505 | 0.130 | 0.130 |
|  | 5 | Cortical | -0.120 | 0.246 | 0.445 |
|  |  | Subcortical | -0.721 | 0.040* | 0.060 |
|  | 10 | Cortical | 0.059 | 0.365 | 0.445 |
|  |  | Subcortical | -0.703 | 0.039* | 0.060 |

**Supplementary Figure 7. ENIGMA-GEDAR correlation.** Figure depicts spearman correlations between ENIGMA maps of structural differences and GEDAR maps calculated at the three different thresholds separately for cortical and subcortical regions. Significance was calculated by generating 1000 random spin permutations to account for spatial autocorrelation after FDR correction for multiple comparisons across disorders. The * highlights significant correlations at p_spin-FDR_<0.05. Abbreviations: Attention Deficit Hyperactivity Disorder (ADHD), Autism Spectrum Disorder (ASD), Anorexia Nervosa (AN), Bipolar Disorder (BD), Major Depressive Disorder (MDD), Obsessive Compulsive Disorder (OCD), Schizophrenia (SCZ).

**
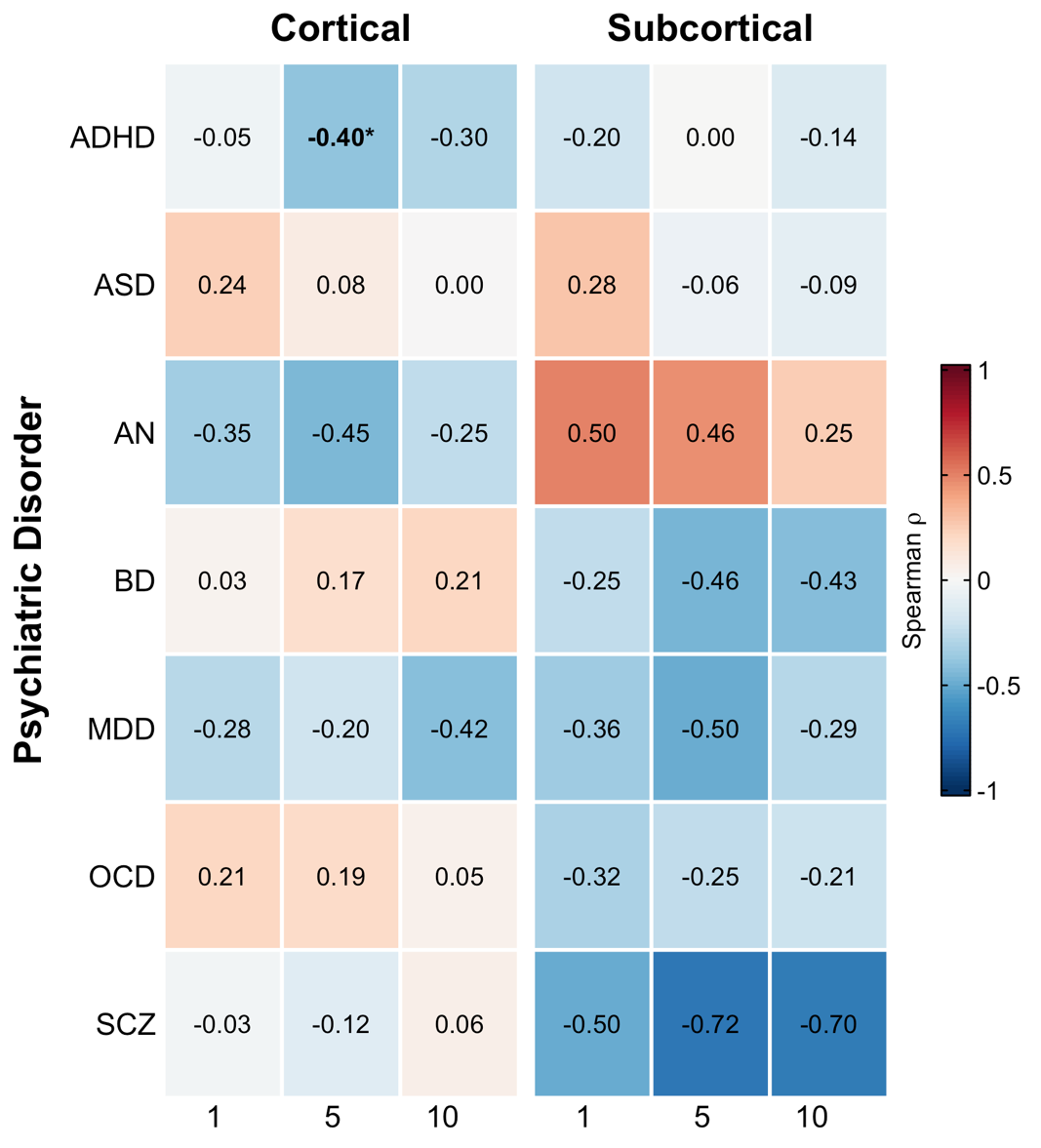
**

**Supplementary Table S4. Correlations between heritability and GEDAR–ENIGMA correlation across disorders. Spearman correlations coefficients were calculated between the middle point of heritability intervals and the estimated GEDAR-ENIGMA correlation. Both refers to GEDARs calculated from up- and down-regulated genes combined, while down- and up-regulated subplots refer to GEDARs calculate with either up- or down-regulated genes only. The * highlights significant correlations at a p<0.05.**

| **Scheme** | **Threshold** | **Compartment** | **Spearman correlation** | **p-value** | **FDR-corrected p-value** |
| --- | --- | --- | --- | --- | --- |
| Both | 1 | Cortical | -0.143 | 0.760 | 0.760 |
|  |  | Subcortical | -0.815 | 0.025* | 0.025* |
|  | 5 | Cortical | -0.036 | 0.939 | 0.939 |
|  |  | Subcortical | -0.429 | 0.337 | 0.337 |
|  | 10 | Cortical | -0.250 | 0.589 | 0.589 |
|  |  | Subcortical | 0.607 | 0.148 | 0.148 |
| Down-regulated | 1 | Cortical | 0.464 | 0.294 | 0.294 |
|  |  | Subcortical | 0.214 | 0.645 | 0.645 |
|  | 5 | Cortical | 0.036 | 0.939 | 0.939 |
|  |  | Subcortical | 0.071 | 0.879 | 0.879 |
|  | 10 | Cortical | 0.357 | 0.432 | 0.432 |
|  |  | Subcortical | 0.036 | 0.939 | 0.939 |
| Up-regulated | 1 | Cortical | -0.929 | 0.003** | 0.003** |
|  |  | Subcortical | -0.643 | 0.119 | 0.119 |
|  | 5 | Cortical | 0.036 | 0.939 | 0.939 |
|  |  | Subcortical | 0.536 | 0.215 | 0.215 |
|  | 10 | Cortical | -0.429 | 0.337 | 0.337 |
|  |  | Subcortical | 0.523 | 0.229 | 0.229 |

**Supplementary Table S5.** The table shows significantly enriched pathways emerging from the pathway enrichment analysis on the lists of genetically-predicted differentially regulated genes from TWAS for those disorders for which we found a significant association between GEDAR and ENIGMA maps.

| **Disorder** | **Threshold (%)** | **Gene Regulation** | **Geneset Source** | **Term ID** | **Term Name** | **p_adj_** |
| --- | --- | --- | --- | --- | --- | --- |
| MDD | 1 | UP | GO:MF | GO:0140311 | Protein sequestering activity | 2.975 x 10^-2^ |
|  |  |  | GO:CC | GO:0042611 | MHC protein complex | 9.378 x 10^-3^ |
| ADHD | 10 | DOWN | GO:BP | GO: 0051960 | Regulation of nervous system development | 1.585 x 10^-2^ |
|  |  |  | GO:BP | GO: 0050767 | Regulation of neurogenesis | 1.651 x 10^-2^ |
|  |  |  | GO:BP | GO: 0051960 | Regulation of multicellular organismal development | 4.499 x 10^-2^ |
|  | 5 | DOWN | GO:CC | GO: 0016342 | Catenin complex | 2.011 x 10^-2^ |
| MDD | 10 | DOWN | GO:CC | GO:0042613 | MHC class II protein complex | 4.166 x 10^-2^ |
|  |  |  | GO:CC | GO:0042611 | MHC protein complex | 8.406 x 10^-5^ |
|  |  |  | GO:MF | GO:0023023 | MHC protein complex binding | 3.242 x 10^-2^ |
|  |  |  | GO:MF | GO:0003823 | Antigen binding | 1.053 x 10^-2^ |
|  |  |  | GO:BP | GO:0019882 | antigen processing and presentation | 6.025 x 10^-4^ |
|  |  |  | GO:BP | GO:0019883 | antigen processing and presentation of endogenous antigen | 4.860 x 10^-2^ |
|  |  |  | GO:BP | GO:0002483 | antigen processing and presentation of endogenous peptide antigen | 1.693 x 10^-2^ |
|  |  |  | GO:BP | GO:0019884 | antigen processing and presentation of exogenous antigen | 1.226 x 10^-2^ |
|  |  |  | GO:BP | GO:0019886 | antigen processing and presentation of exogenous peptide antigen via MHC class II | 3.205 x 10^-2^ |
|  |  |  | GO:BP | GO:0048002 | antigen processing and presentation of peptide antigen | 2.126 x 10^-4^ |
|  |  |  | GO:BP | GO:0007155 | cell adhesion | 2.523 x 10^-2^ |
|  |  |  | GO:CC | GO:0071944 | cell periphery | 1.216 x 10^-6^ |
|  |  |  | GO:CC | GO:0009986 | cell surface | 1.192 x 10^-4^ |
|  |  |  | GO:CC | GO:0062023 | collagen-containing extracellular matrix | 1.496 x 10^-2^ |
|  |  |  | GO:CC | GO:0030312 | external encapsulating structure | 3.817 x 10^-2^ |
|  |  |  | GO:CC | GO:0009897 | external side of plasma membrane | 1.006 x 10^-3^ |
|  |  |  | GO:CC | GO:0031012 | extracellular matrix | 3.754 x 10^-2^ |
|  |  |  | GO:BP | GO:0002768 | immune response-regulating cell surface receptor signaling pathway | 3.982 x 10^-2^ |
|  |  |  | GO:CC | GO:0098553 | lumenal side of endoplasmic reticulum membrane | 4.329 x 10^-3^ |
|  |  |  | GO:CC | GO:0098576 | lumenal side of membrane | 1.917 x 10^-2^ |
|  |  |  | GO:CC | GO:0016020 | membrane | 3.789 x 10^-4^ |
|  |  |  | GO:MF | GO:0060089 | molecular transducer activity | 1.764 x 10^-2^ |
|  |  |  | GO:MF | GO:0042605 | peptide antigen binding | 1.583 x 10^-4^ |
|  |  |  | GO:MF | GO:0042277 | peptide binding | 2.378 x 10^-2^ |
|  |  |  | GO:CC | GO:0005886 | plasma membrane | 4.688 x 10^-5^ |
|  |  |  | GO:BP | GO:1902533 | positive regulation of intracellular signal transduction | 3.090 x 10^-2^ |
|  |  |  | GO:BP | GO:0048584 | positive regulation of response to stimulus | 2.011 x 10^-2^ |
|  |  |  | GO:BP | GO:0097104 | postsynaptic membrane assembly | 3.615 x 10^-2^ |
|  |  |  | GO:BP | GO:0002822 | regulation of adaptive immune response based on somatic recombination of immune receptors built from immunoglobulin superfamily domains | 2.325 x 10^-2^ |
|  |  |  | GO:BP | GO:0050770 | regulation of axonogenesis | 6.242 x 10^-3^ |
|  |  |  | GO:BP | GO:0050776 | regulation of immune response | 6.290 x 10^-3^ |
|  |  |  | GO:BP | GO:0048583 | regulation of response to stimulus | 4.235 x 10^-2^ |
|  |  |  | GO:CC | GO:0098552 | side of membrane | 8.083 x 10^-3^ |
|  |  |  | GO:MF | GO:0038023 | signaling receptor activity | 1.764 x 10^-2^ |
| SCZ | 10 | DOWN | GO:MF | GO:0042605 | peptide antigen binding | 3.65 x 10^-6^ |
|  | 5 |  |  |  |  | 2.60 x 10^-6^ |
|  | 10 |  | GO:MF | GO:0046977 | TAP binding | 3.999 x 10^-3^ |
|  | 10 |  | GO:MF | GO:0042277 | peptide binding | 1.657 x 10^-2^ |
|  | 5 |  |  |  |  | 8.399 x 10^-3^ |
|  | 10 |  | GO:MF | GO:0003823 | antigen binding | 5.957 x 10^-2^ |
|  | 5 |  |  |  |  | 1.029 x 10^-2^ |
|  | 5 |  | GO:MF | GO:0023026 | MHC class II protein complex binding | 3.000 x 10^-2^ |
|  | 10 |  | GO:BP | GO:0048002 | antigen processing and presentation of peptide antigen | 3.174 x 10^-7^ |
|  | 5 |  |  |  |  | 2.220 x 10^-6^ |
|  | 10 |  | GO:BP | GO:0019882 | antigen processing and presentation | 3.898 x 10^-5^ |
|  | 5 |  |  |  |  | 9.565 x 10^-5^ |
|  | 10 |  | GO:BP | GO:0002476 | antigen processing and presentation of endogenous peptide antigen via MHC class Ib | 2.264 x 10^-3^ |
|  | 5 |  |  |  |  | 2.085 x 10^-2^ |
|  | 10 |  | GO:BP | GO:0002484 | antigen processing and presentation of endogenous peptide antigen via MHC class I via ER pathway | 2.264 x 10^-3^ |
|  | 5 |  |  |  |  | 2.085 x 10^-2^ |
|  | 10 |  | GO:BP | GO:0019886 | antigen processing and presentation of exogenous peptide antigen via MHC class II | 3.205 x 10^-2^ |
|  | 5 |  |  |  |  | 2.563 x 10^-3^ |
|  | 10 |  | GO:BP | GO:0002822 | regulation of adaptive immune response based on somatic recombination of immune receptors built from immunoglobulin superfamily domains | 2.325 x 10^-2^ |
|  | 5 |  |  |  |  | 2.650 x 10^-3^ |
|  | 5 |  | GO:BP | GO:0002250 | adaptive immune response | 2.938 x 10^-3^ |
|  | 10 |  | GO:BP | GO:0002428 | antigen processing and presentation of peptide antigen via MHC class Ib | 3.005 x 10^-3^ |
|  | 5 |  |  |  |  | 2.561 x 10^-2^ |
|  | 10 |  | GO:BP | GO:0002474 | antigen processing and presentation of peptide antigen via MHC class I | 3.170 x 10^-3^ |
|  | 10 |  | GO:BP | GO:0002824 | positive regulation of adaptive immune response based on somatic recombination of immune receptors built from immunoglobulin superfamily domains | 1.200 x 10^-2^ |
|  | 5 |  |  |  |  | 3.214 x 10^-3^ |
|  | 10 |  | GO:BP | GO:0002478 | antigen processing and presentation of exogenous peptide antigen | 4.183 x 10^-3^ |
|  | 5 |  |  |  |  | 9.083 x 10^-3^ |
|  | 10 |  | GO:BP | GO:0002821 | positive regulation of adaptive immune response | 1.641 x 10^-2^ |
|  | 5 |  |  |  |  | 4.244 x 10^-3^ |
|  | 10 |  | GO:BP | GO:0002819 | regulation of adaptive immune response | 4.032 x 10^-2^ |
|  | 5 |  |  |  |  | 4.407 x 10^-3^ |
|  | 5 |  | GO:BP | GO:0002495 | antigen processing and presentation of peptide antigen via MHC class II | 4.462 x 10^-3^ |
|  | 5 |  | GO:BP | GO:0002504 | antigen processing and presentation of peptide or polysaccharide antigen via MHC class II | 7.425 x 10^-3^ |
|  | 10 |  | GO:BP | GO:0051251 | positive regulation of lymphocyte activation | 3.070 x 10^-2^ |
|  | 5 |  |  |  |  | 9.227 x 10^-3^ |
|  | 10 |  | GO:BP | GO:0019885 | antigen processing and presentation of endogenous peptide antigen via MHC class I | 1.176 x 10^-2^ |
|  | 10 |  | GO:BP | GO:0019884 | antigen processing and presentation of exogenous antigen | 1.226 x 10^-2^ |
|  | 5 |  |  |  |  | 2.141 x 10^-2^ |
|  | 10 |  | GO:BP | GO:0001914 | regulation of T cell mediated cytotoxicity | 1.226 x 10^-2^ |
|  | 5 |  |  |  |  | 2.141 x 10^-2^ |
|  | 5 |  | GO:BP | GO:0002399 | MHC class II protein complex assembly | 1.317 x 10^-2^ |
|  | 5 |  | GO:BP | GO:0002503 | peptide antigen assembly with MHC class II protein complex | 1.317 x 10^-2^ |
|  | 10 |  | GO:BP | GO:0002475 | antigen processing and presentation via MHC class Ib | 1.417 x 10^-2^ |
|  | 10 |  | GO:BP | GO:0050870 | positive regulation of T cell activation | 2.291 x 10^-2^ |
|  | 5 |  |  |  |  | 1.593 x 10^-2^ |
|  | 5 |  | GO:BP | GO:0050778 | positive regulation of immune response | 1.642 x 10^-2^ |
|  | 5 |  | GO:BP | GO:0002486 | antigen processing and presentation of endogenous peptide antigen via MHC class I via ER pathway, TAP-independent | 1.672 x 10^-2^ |
|  | 10 |  | GO:BP | GO:0002483 | antigen processing and presentation of endogenous peptide antigen | 1.693 x 10^-2^ |
|  | 5 |  | GO:BP | GO:0002696 | positive regulation of leukocyte activation | 1.799 x 10^-2^ |
|  | 5 |  | GO:BP | GO:0050776 | regulation of immune response | 1.967 x 10^-2^ |
|  | 5 |  | GO:BP | GO:0002684 | positive regulation of immune system process | 2.148 x 10^-2^ |
|  | 5 |  | GO:BP | GO:0050867 | positive regulation of cell activation | 2.524 x 10^-2^ |
|  | 10 |  | GO:BP | GO:0001913 | T cell mediated cytotoxicity | 2.681 x 10^-2^ |
|  | 5 |  |  |  |  | 4.022 x 10^-2^ |
|  | 10 |  | GO:BP | GO:0022409 | positive regulation of cell-cell adhesion | 2.686 x 10^-2^ |
|  | 5 |  | GO:BP | GO:0002460 | adaptive immune response based on somatic recombination of immune receptors built from immunoglobulin superfamily domains | 2.727 x 10^-2^ |
|  | 5 |  | GO:BP | GO:0051249 | regulation of lymphocyte activation | 2.757 x 10^-2^ |
|  | 10 |  | GO:BP | GO:1903039 | positive regulation of leukocyte cell-cell adhesion | 4.616 x 10^-2^ |
|  | 5 |  |  |  |  | 2.831 x 10^-2^ |
|  | 10 |  | GO:BP | GO:0050890 | cognition | 3.070 x 10^-2^ |
|  | 10 |  | GO:BP | GO:1902105 | regulation of leukocyte differentiation | 3.153 x 10^-2^ |
|  | 10 |  | GO:BP | GO:0051240 | positive regulation of multicellular organismal process | 3.179 x 10^-2^ |
|  | 5 |  | GO:BP | GO:0002501 | peptide antigen assembly with MHC protein complex | 3.717 x 10^-2^ |
|  | 5 |  | GO:BP | GO:0002396 | MHC protein complex assembly | 3.717 x 10^-2^ |
|  | 10 |  | GO:BP | GO:0048583 | regulation of response to stimulus | 4.235 x 10^-2^ |
|  | 10 |  | GO:BP | GO:0019883 | antigen processing and presentation of endogenous antigen | 4.860 x 10^-2^ |
|  | 10 |  | GO:CC | GO:0042611 | MHC protein complex | 8.730 x 10^-5^ |
|  | 5 |  |  |  |  | 2.358 x 10^-6^ |
|  | 10 |  | GO:CC | GO:0042613 | MHC class II protein complex | 4.196 x 10^-2^ |
|  | 5 |  |  |  |  | 4.638 x 10^-3^ |
|  | 5 |  | GO:CC | GO:0071944 | cell periphery | 8.410 x 10^-3^ |
|  | 10 |  | GO:CC | GO:0005886 | plasma membrane | 2.881 x 10^-2^ |
|  | 5 |  |  |  |  | 1.115 x 10^-2^ |
|  | 5 |  | GO:CC | GO:0098553 | lumenal side of endoplasmic reticulum membrane | 1.542 x 10^-2^ |
|  | 5 |  | GO:CC | GO:0098576 | lumenal side of membrane | 4.674 x 10^-2^ |
